# Anterior subcutaneous internal fixator (INFIX) versus plate fixation for pelvic anterior ring fracture

**DOI:** 10.1038/s41598-019-39068-7

**Published:** 2019-02-22

**Authors:** Yingchao Yin, Junhao Luo, Ruipeng Zhang, Shilun Li, Zhenqing Jiao, Yingze Zhang, Zhiyong Hou

**Affiliations:** 1 Department of Orthopaedic Surgery, the Third Hospital of Hebei Medical University, Shijiazhuang, Hebei 050051 P. R. China; 2 Key laboratory of biomechanics of Hebei Province, Shijiazhuang, Hebei 050051 P. R. China; 3 Chinese Academy of Engineering, Beijing, 100088 P. R. China

## Abstract

The aim of this study was to compare the clinical outcomes in patients with unstable anterior pelvic ring fractures after treatment with anterior subcutaneous internal fixator (INFIX) or plate fixation. We performed a retrospective study from August 2015 to October 2017. A consecutive series of 74 patients who underwent surgical treatment of their anterior pelvic ring (35 treated with INFIX and 39 treated with plates) were studied. Data collected included patients’ demographic data, injury severity score (ISS), AO/OTA classification, injury mechanism, time to surgery, procedure time, and blood loss. The quality of postoperative reduction were assessed by postoperative radiographs using the Tornetta and Matta method. Functional outcome was evaluated using Majeed scoring system. In the INFIX group, ten patients developed LFCN paralysis, one patient suffered from superficial infection. Three screw loosening cases and two wound infection cases occurred in the plate group. INFIX is relatively minimally invasive and time-saving than the reconstruction plate in the treatment of anterior pelvic ring fracture. However, plate fixation increases the rate of anatomic reduction of the pelvic anterior ring fracture. Plates also provide a higher functional outcome compared with INFIX. INFIX is especially suitable in patients with urological injury, which can also decrease the wound infection rate.

## Introduction

Unstable pelvic ring fractures are usually associated with high energy trauma. They account for about 1.5–3.9% of all fractures^1^, but they have a high rate of morbidity and mortality. Although the posterior pelvic ring provides the main stability (60%), the anterior ring still account for 40% of stability^2^. Antero-posterior compression, lateral compression or vertical shear injury of the pelvis (AO/OTA B1–3, C1–3) require fixation of the anterior ring or anterior-posterior ring simultaneously^3^.

The external fixator is the most widely used treatment for initial and temporary stabilization of anterior pelvic ring injury, especially in emergency situations^4^. It can be quickly placed and can easily stabilize the disrupted pelvic ring and decrease pelvic cavity hemorrhage. However, many clinical complications associated with the external fixator have been reported, including wound infection, loosening of the fixator, and impingement on the skin^5,6^. Moreover, the anterior pelvic external fixator limits patients’ daily activities, such as sitting, lying in the lateral position, rolling over, and sexual intercourse.

Recently, anterior subcutaneous internal fixator (INFIX) was proposed by several scholars to treat anterior pelvic ring injury^7,8^. INFIX was invented based upon the same biomechanical principle as the traditional external fixator, but it is placed subcutaneously. It proved to be stiffer than the traditional external fixator, and at the same time eliminates the open pin tracts, which increased the infection rate and nursing care. INFIX was initially designed as an alternative to the external fixator, but recently its indications have been expanded and multiple complications have been reported^9–15^.

Few reports have compared the outcome of INFIX with anterior plate fixation. The purpose of this retrospective study was to compare INFIX with plate fixation for anterior pelvic ring injuries by assessing radiological reductions, functional outcomes and related complications.

## Patients and Methods

This study was approved by the Institutional Review Board of Hebei Medical University, Shijiazhuang, China. All institutional guidelines for the care and treatment of patients were rigorously followed. Informed consents was obtained from all individual participants included in the study.

A retrospective review of anterior pelvic ring fracture was performed from August 2015 to October 2017. Inclusion criteria was unstable pelvic fracture evaluated by the senior trauma surgeon, which required anterior fixation after confirming that the posterior ring was stable (either because the posterior ring was intact or recovered after fixation). The exclusion criteria included age <18 years, open fracture with contaminated wound, and pathological fracture. Patients’ pelvises were evaluated on preoperative radiography (including anterior-posterior (AP), inlet, and outlet views) and CT. Two experienced orthopedists evaluated the imaging data and classified each fracture using the AO Foundation and Orthopaedic Trauma Association (OTA/AO) classification^3^.

The following information was recorded: age, gender, injury severity score (ISS), AO/OTA fracture classification, mechanism of injury, time to pelvic surgery, procedure time and blood loss. Postoperative radiological assessment, most recent follow-up, functional outcome scores, and complications were used to evaluate the effect of operation.

### Surgical procedure

The patient was positioned supine on a radiolucent operating table. All patients received general anesthesia. Posterior pelvic ring injury was addressed as the priority of fixation if needed. Anterior ring fixation was performed after evaluating the stabilization of the posterior ring.

**Figure 1:**
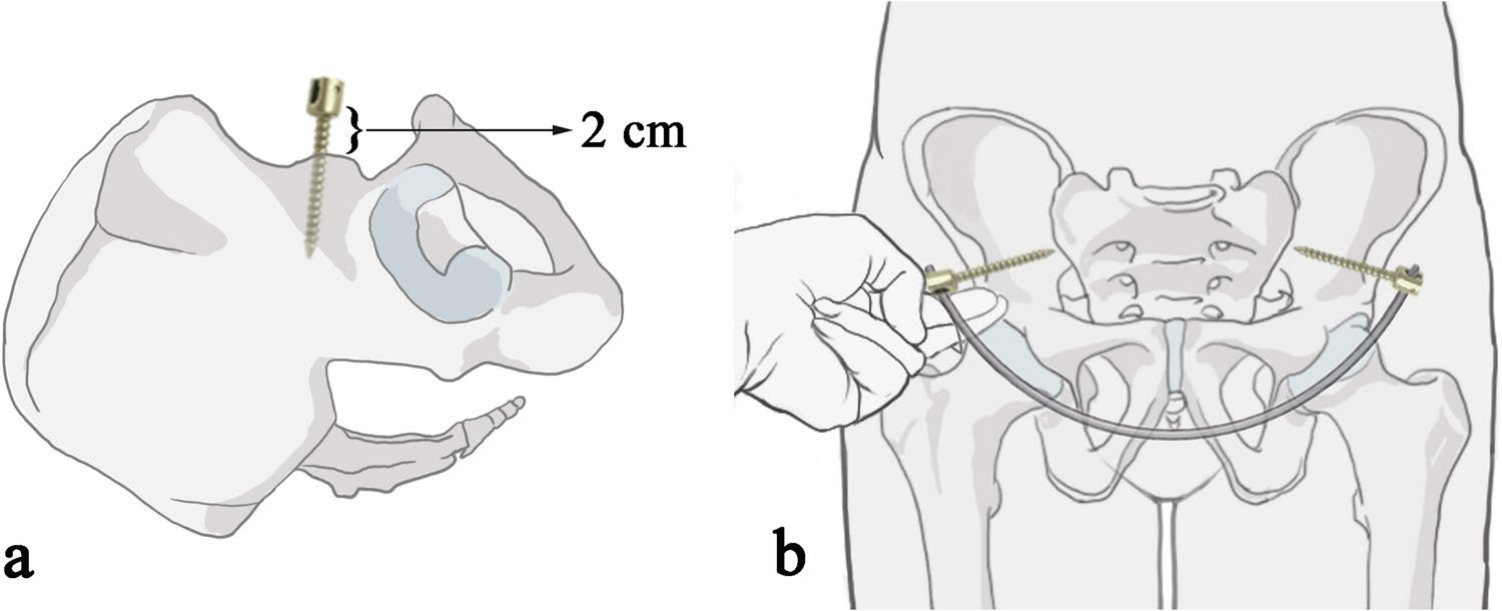
(**a**) The pedicle screw head should be kept at least 2 cm from the bone surface to avoid compression of the vascular tissue after installation of the connecting rod. (**b**) After installing the connectting rod, the overlapped index finger and the middle finger were used to check if there was enough space between the connecting rod and bone.

**Figure 2:**
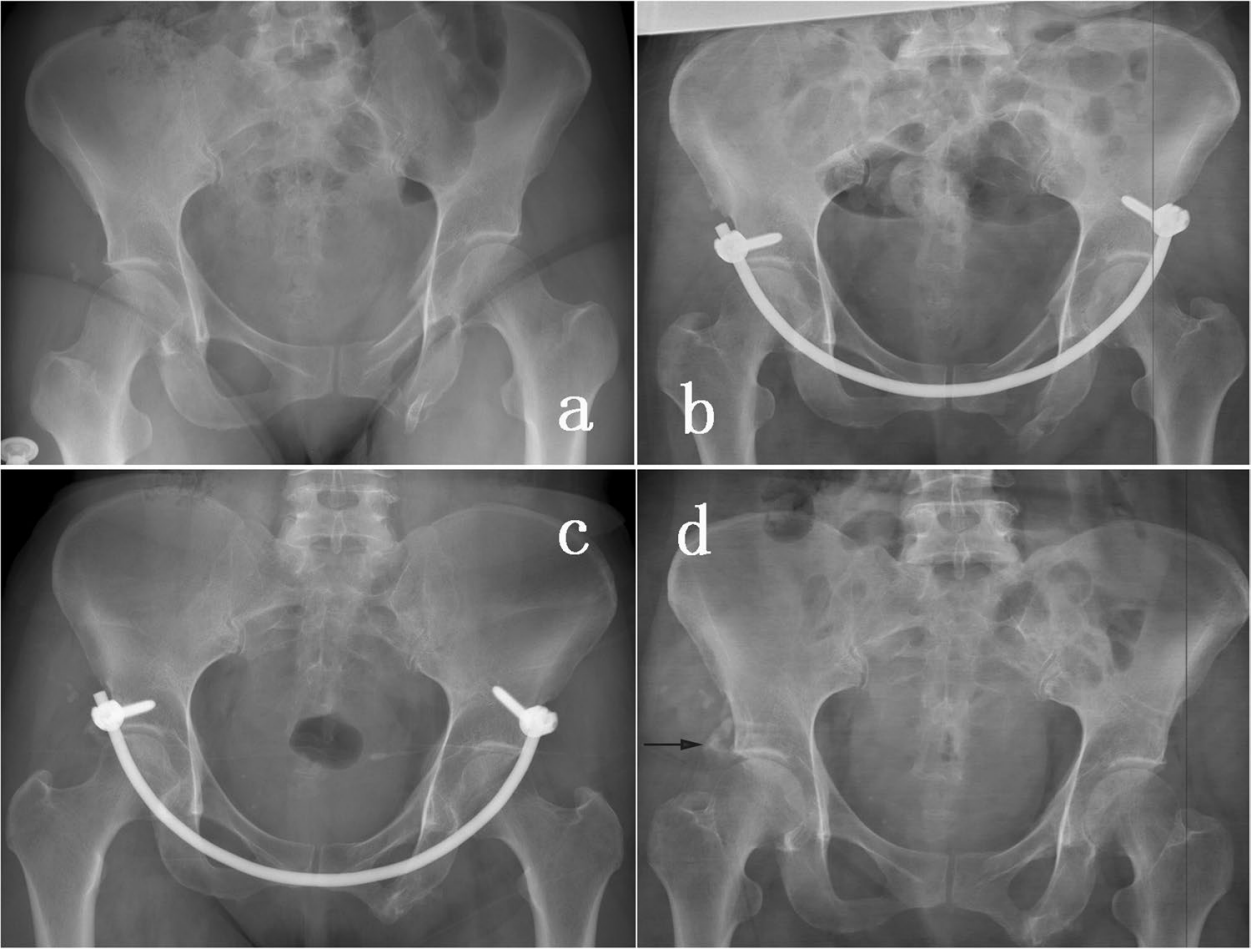
(**a**) AO/OTA 61-B2 pelvic ring injury. (**b**) Postoperative pelvic AP view. A “distraction force” was performed during locking the second pedicle screw end cap. (**c**) Six months follow-up AP view x-ray. (**d**) Pelvic AP view after removal of the INFIX. The black arrow indicates heterotopic ossification.

In the INFIX group, a 2–3 cm oblique incision was made with the anterior inferior iliac spine (AIIS) as the center. The intermuscle space between the tensor fascia lata and sartorius was bluntly separated to expose the AIIS. Two Hohhman retractors were used to protect the soft tissue. A starting point was created using the awl between the medial and lateral bony plates of the ilium. A polyaxial pedicle screw (6.5-mm diameter, 50-mm long) was inserted from the entrance. The pedicle screw head was kept at least 2 cm from the bone surface to avoid compression of the vascular tissue after installation of the connecting rod (Fig. 1a). The same procedure was performed at the contralateral side of AIIS. A subcutaneous tunnel was made from one AIIS incision towards the other side over the deep fascia. A curved titanium rod (5.5-mm diameter) was inserted via the subcutaneous tunnel to connect the bilateral pedicle screws. Both sides of the end cap was tighten by the screwdriver. After that, two overlapping fingers were used to check if there was enough space between the rod and bone (Fig. 1b). A typical case is shown in Fig. 2.

**Figure 3:**
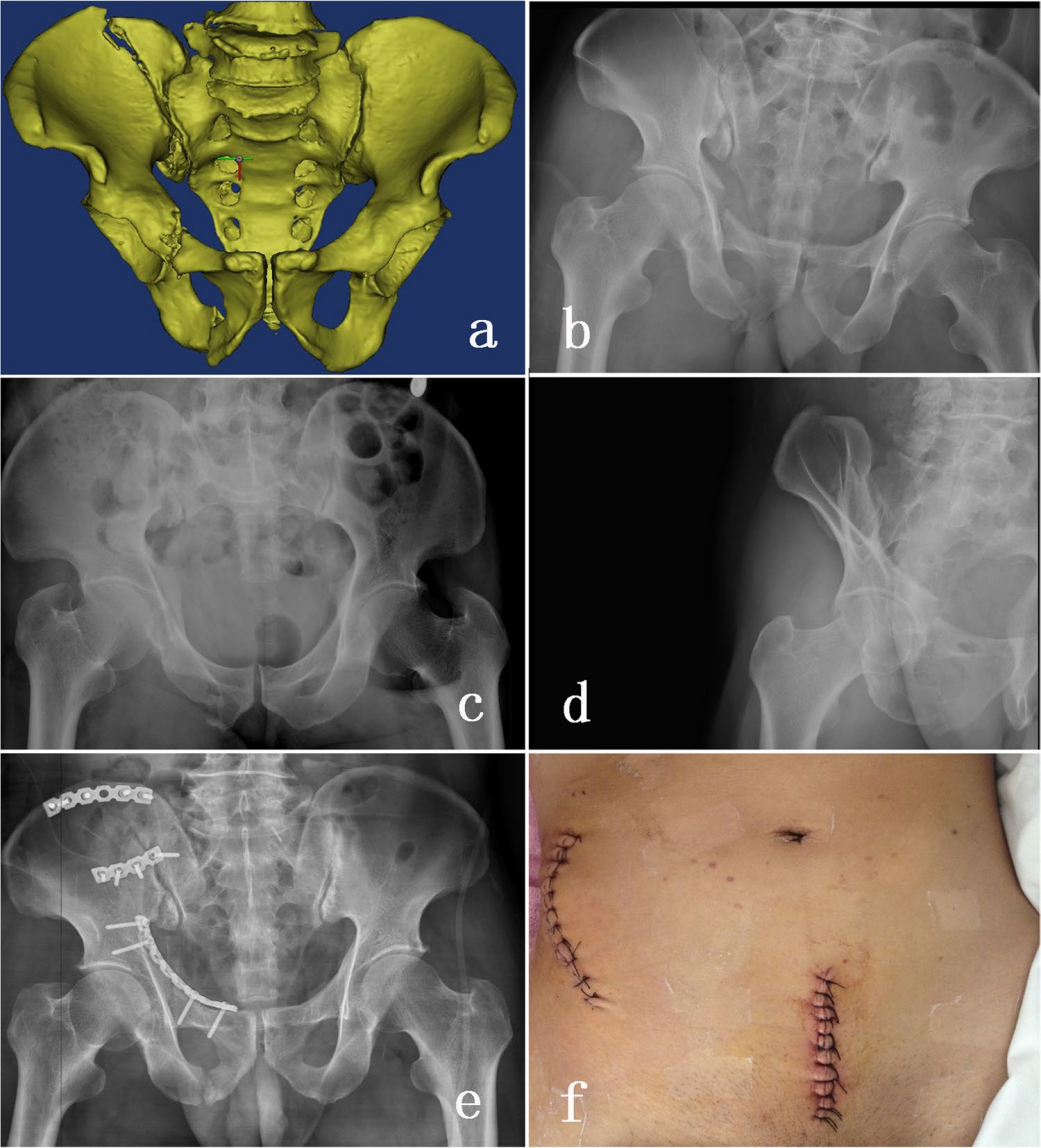
(**a**) Preoperative three-dimensional computed tomography reconstruction. (**b**) Pelvic AP view of an AO/OTA 61-C1 pelvic ring injury. (**c**,**d**) Pelvic inlet view and obturator oblique view showed the right pubic rami and the right iliac wing fracture. (**e**) The Stoppa approach combined with the iliac fossa approach were used to fix the fractures. (**f**) Postoperative imaging of the surgical wounds.

In the plate group, the anterior plate was placed through modified Stoppa approach. The iliac fossa approach was used if the iliac crest fracture existed. A 10–15 cm horizontal or vertical incision was performed 1–2 cm superior to pubic symphysis. The linea alba split in a longitudinal manner. The rectus abdominis muscles were retracted laterally without sharp dissection. We mobilized the peritoneum and the bladder with a wider retractor. Then, the pelvic anterior ring and quadrilateral surface was exposed through the Stoppa approach. The pre-bending reconstructive plate (3.5-mm) was placed at the medial side of the superior pubic rami and pelvic brim to stabilize the fragments. A typical case is shown in Fig. 3.

### Radiographic displacement measurement

Reductions of the anterior pelvic ring were assessed immediately using the postoperative X-ray. The largest anterior displacement of the pelvis was measured in three standard radiographs (AP, inlet and outlet view). Grading was performed according to the method of Tornetta and Matta^16^. The reduction quality was graded as excellent (displacement was within 4 mm), good (5 to 10 mm), fair (10 to 20 mm), or poor (displacement over 20 mm). For the postoperative reduction quality, excellent and good were regarded as satisfactory results in this study.

### Postoperative rehabilitation and follow-up

The postoperative regimens were similar between groups. Patients were encouraged to train their lower limbs on the bed if the pain could be tolerated, postoperatively. Postoperative partial-weightbearing was permitted at eight weeks for AO/OTA type B injuries and 12 weeks for type C injuries. Full weight-bearing was permitted if the fracture healing was confirmed with radiographs.

Majeed rating scale was used to evaluate the functional outcome^17^. Patients were followed up by phone or at the clinic. The functional recovery was scored by asking the rating scale questions. These questions included the subjective feelings of pain, return to the work, the feelings of sitting, sexual intercourse, standing, walking distance, and the condition of gait. The aggregate score was classified as excellent (>85), good (70–84), fair (55–69), or poor (<55).

### Statistical analysis

The distributions of all variables were evaluated for normality by using the Shapiro-Wilk test. Data satisfying normality were presented as the mean and standard deviation. Data that did not meet normality were presented as medians and quartiles. The difference in gender distribution, fracture AO/OTA classification and injury mechanism between the two groups was determined using the Chi-squared test. The Student *t*-test and nonparametric test were used to analyze the continuous variables. All statistical analyses were performed using IBM SPSS Statistics for Windows, version 21.0 (IBM, Armonk, NY, USA). A value of *p* less than 0.05 was considered significant.

## Results

Above all, 74 patients with anterior pelvic ring injuries, treated with INFIX or plates at our department, were included in this study. Among them, 35 patients were treated with INFIX and the other 39 patients were fixed with plates. The average age and gender distribution between these two groups was not statistically significant (*p* > 0.05). These two groups were similar in ISS and OTA classifications, as well as their mechanism of injury and time to pelvic surgery (*p* > 0.05) (Table 1). The INFIX group was superior to the plate group in terms of procedure time and blood loss (*p* < 0.05).

**Table 1:** Patient Demographics of two groups (ISS, injury severity score).

Parameter	INFIX group (n = 35)	Plate group (n = 39)	*p*
Age (yrs)	41.7 ± 12.6	43.6 ± 13.2	0.517
Gender: male/female	19/16	24/15	0.528
ISS	29 (24, 34)	24 (21, 29)	0.329
AO/OTA Classification			0.442
61-B			
61-B1	6	9	
61-B2	12	11	
61-B3	7	5	
61-C			
61-C1	4	10	
61-C2	6	4	
61-C3	0	0	
Injury mechanism			0.469
Fall from height	7	8	
Traffic accident	18	15	
Other	10	16	
Time to surgery (d)	7 (5, 9)	6 (4, 9)	0.107
Procedure time (min)	70.3 ± 11.8	98.7 ± 17.3	0.000
Blood loss (ml)	97.1 ± 15.6	449.2 ± 214.1	0.000

**Figure 4:**
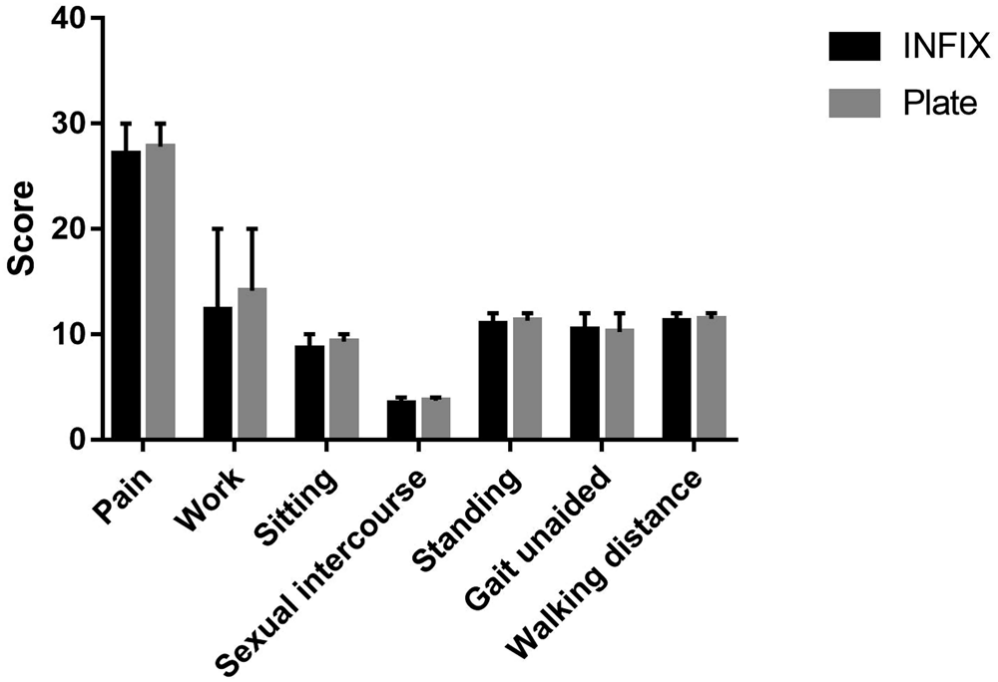
The comparison of each Majeed item between the two groups.

All patients’ fractures healed without incident. Postoperative radiographic reduction grading shows that the two groups had a similar satisfactory rate ((“Excellent” + “Good”)/total number of patients) (74.29% *vs*. 79.49%, *p* = 0.595). However, the plate group achieved more anatomic restoration (“Excellent”) of the anterior ring (*p* = 0.019). The median follow-up was 27 months (range 21–32) in the INFIX group and 23 months (range 17–33) in the plate group (*p* = 0.248). The plate group received a higher Majeed rating which was statistically significant (*p* = 0.029) (Table 2). However, no statistically significant difference was found in the individual items of the Majeed scores (Table 2, Fig. 4).

**Table 2:** Postoperative radiology and functional outcome grading.

	INFIX (n = 35)	plating (n = 39)	*p*
Tornetta and Matta grading			0.019
*Excellent*	7	21	
*Good*	19	10	
*Fair*	6	6	
*Poor*	3	2	
Satisfactory rate	26/35, (74.29%)	31/39, (79.49%)	0.595
Follow up time(month)	27 (21, 32)	23 (17, 33)	0.248
Majeed score	84.14 ± 8.07	88.05 ± 7.01	0.029
*Pain*	25 (25, 30)	30 (25, 30)	0.233
*Work*	12 (8, 16)	12 (12, 20)	0.069
*Sitting*	10 (8, 10)	10 (8, 10)	0.053
*Sexual intercourse*	4 (3, 4)	4 (4, 4)	0.243
*Standing*	12 (10, 12)	12 (10, 12)	0.281
*Gait unaided*	10 (10, 12)	10 (10, 12)	0.654
*Walking distance*	12 (10, 12)	12 (10, 12)	0.289

### Complications

Ten (28.57%) patients who underwent INFIX placement suffered LFCN paralysis. Its symptoms mainly manifested as anterolateral skin numbness of the affected thigh. Nearly one-third of the ten patients recovered immediately after the device was removed. Moreover, the remaining two-thirds of patients stated that their symptoms gradually disappeared within three months after its removal. Twelve (34.29%) patients developed heterotopic ossification; however, all of them were asymptomatic. One superficial infection occurred because of the poor soft tissue condition at the surgical site; this patients’ condition was resolved using oral antibiotics.

In the plate group, 5 out of the 39 patients developed asymptomatic heterotopic ossification. Additionally, three cases who underwent plate fixation experienced screw loosening. Infections in the plate group occurred in 2 patients with bladder or urethral disruptions. Both patients achieved wound healing after debridement and continuous dressing changes.

## Discussion

Treatment of unstable pelvic fractures often requires fixation of both anterior and posterior pelvic rings. The pelvic posterior ring is frequently fixed with plates^18^, iliosacral screws^19^ or lumbo-sacro-iliac screws^20^. Using reconstruction plates is a traditional fixator, which provides excellent stability for fixation of the anterior ring fracture^21^. The appearance of INFIX provides a new alternative treatment^7,8^. The most important finding of this study was that INFIX is more minimally invasive and time-saving for the treatment of anterior pelvic ring injuries, but that plates can provide a higher anatomical reduction rate.

The external fixator is an effective tool for anterior ring fixation and has been widely used. However, it is associated with many complications, such as pin-track infection^22^, aseptic loosening^23^ and loss of reduction^24^. In 2009, Kuttner *et al*. first reported using the pedicle screw-rod system to fixate anterior ring fractures in 19 patients^25^. Over the next few years, more researchers explored the stability, indications and complications of INFIX through biomechanical and clinical studies^7–9,12,26^. INFIX has its unique advantages, but still was reported to have several complications^11,12,27^.

Vaidya *et al*. performed a study to compare INFIX with plates in the treatment of symphysis disruption^28^. The results demonstrated that INFIX achieves a better outcome in reducing the symphyseal widening. In this study, the plate group received a greater anatomical reduction in the treatment of anterior ring injury. The main reason being INFIX only performs closed reduction of the upper and lower pubic rami fracture. However, the current study showed that blood loss of the plate group was 4.5-fold than that of INFIX group approximately. The main reason for this is INFIX can be implanted subcutaneously. And the plate group needs exposing the fracture site and reduce the fracture to original anatomy. The blood vessels around the pelvis are rich, and the peeling of soft tissue and the exposure process of the fracture ends lead to more intraoperative bleeding. Intra-operative blood cell salvage device was recommended for the complex pelvic fracture according to the previous study^29,30^.

INFIX is more appropriate to be used in less displacement fracture patients, especially obesity or urethral injury patients^28^. Since the anterior ring fracture was not exposed when placing the INFIX, the technique of closed reduction is particularly important. Our closing reduction experiences are summarized as follows: For displaced fracture type of anterior pelvic ring fractures (APC-Type in Young-Burgess classification), firstly, one side of the rod should be locked by pedicle screw end cap, then the other side of the rod, under a lateral compression of the pelvis, should be locked. For overlapped fracture type of anterior ring fractures (LC-Type in Young-Burgess classification), a distraction force should be performed while locking the second pedicle screw end cap.

In recent years, plate fixation for pelvic anterior ring fractures is often performed using the modified Stoppa approach^31,32^. This is a less invasive approach that provides a good exposure of the entire pelvic brim, from the pubic symphysis to the iliosacral joint^33^. Another advantage of this approach is that, with a single incision, it allows fixation of the bilateral pubic rami. An excellent reduction rate of 53.85% was obtained in this study through the Stoppa approach. One new modified technique has been proposed based on the INFIX system, which adds one or two pedicle screws into the pubic tubercle via an invasive incision^15^. This improvement was thought to be capable of restricting the movement of the pubis fragment and affords more stabilization. In this study, the Majeed score was higher in the plate fixation group than in the INFIX group. However, we were unable to find a significant difference in each item of the Majeed score between the two groups.

The main complication that appeared in the INFIX group was LFCN paresthesia. Approximately one third of patients developed the symptom of numbness in the anterolateral side of the affected thigh. All these patients experienced full recovery after 3 months of the implant removal. Hoskins *et al*. reported an LFCN paralysis rate of 57% in their study^27^, which is much higher than other studies. To obtain a more stable fixation, 10-mm diameter pedicle screws were used in their study. The larger screw head maybe contributed to traction LFCN paralysis. In this study, 6.5 mm diameter pedicle screws were used in the INFIX system, which might be a reason for a relatively low rate of neuronal symptoms. Future biomechanical studies are necessary to investigate the optimum diameter than can provide sufficient stability. The pedicle screws were not recommended to be placed too deep to prevent compressing the underlying structures. We recommend using the overlapping index and middle fingers (as showed in Fig. 2) to measure the space between the rod and bone. Meanwhile, the end of the screw should not exceed the area of the anterior superior iliac spine to prevent the formation of protrusions, which may irritate the skin. Asymptomatic heterotopic ossification is the second most common complication reported by previous studies^11,27^, which will not affect the hip function or cause any symptoms. Despite all this, prophylactic treatment, such as radiation therapy or NSAIDs (Non-Steroidal Anti-inflammatory Drugs), should be considered to prevent this complication^34^.

Vaidya *et al*. conducted a biomechanical study to determine stability between monoaxial pedicle screws and polyaxial screws in the application of the INFIX system^35^. Their study declared that polyaxial screws present “favorably” in stiffness testing as compared to the monoaxial screw system and external fixation. Eagan *et al*. tested the INFIX system and traditional external fixation on a posterior ring totally removed synthetic bone model^36^. They concluded that polyaxial INFIX was weaker than the traditional external fixator. But, this situation is extremely rare in the clinic, as the posterior ring accounts for about 60% of pelvic stability^2^. In our study, the polyaxial pedicle screws were used to fixate the anterior ring injury after the posterior ring was stabilized. No reduction loss cases occurred in the INFIX group. Though the polyaxial screw provided inferior stabilization compared to the monoaxial screw, it was enough to fix the anterior pelvic ring.

The limitations of this study included the following points: Firstly, the sample size of this study was relatively small, which may not be representative of all anterior ring pelvic injuries; Secondly, this was a single-center retrospective study, a multi-center prospective studies should be conducted to compare these two fixators.

## Conclusion

INFIX is relatively minimally invasive and time-saving than the reconstruction plate for the treatment of anterior pelvic ring fractures. Plate fixation is recommended for pelvic anterior ring injury patients with obvious displacement. INFIX is especially suitable for anterior ring injury patients with pelvic organ injuries. A higher functional outcome score was obtained by the plate fixation group in the treatment of anterior pelvic ring fractures.
